# Editorial: From Whole-Cell to Single Synapse Engrams - Breaking the Code for Memory Formation, Storage and Recall

**DOI:** 10.3389/fnmol.2022.845516

**Published:** 2022-03-01

**Authors:** Antonino Cattaneo, Marco Mainardi

**Affiliations:** ^1^Laboratory of Biology “Bio@SNS”, Scuola Normale Superiore, Pisa, Italy; ^2^Neuroscience Institute, National Research Council, Pisa, Italy

**Keywords:** memory engrams, synaptic plasticity, learning, dendritic spines, long-term potentiation

The capability of animal brains to process, store, and recall specific information is essential to express acquired survival habits, such as repeating the same path to reach a food-rich area, or to keep out of predators. In Man, the emergence of language and culture have pushed these functions up to defining our individual and collective identities. The rooting of our self in memory is, of course, epitomized by Alzheimer's disease, in which impaired memory formation and recall deprive an individual of its ties, progressively cutting all the bridges with past experience.

Understanding the phenomenology and mechanisms of information processing has been a central problem from the very foundations of modern neuroscience (Sherrington, 1906; Adrian, 1947; Hebb, 1949). Currently, the study of memory gravitates around engrams, i.e., the cellular substrates necessary and sufficient for memory storage and recall (Josselyn and Tonegawa, 2020). Notably, the multiplicity of conceptual and operative definitions about the different modalities, phases and processes associated with memory can be confusing: the Perspective by Hardt and Sossin puts some much needed order on this matter.

Memory engrams are currently conceived as cell assemblies, with their whole-neuron spatial scale being dictated also by limiting technical and experimental reasons. However, decades of studies have shown that the neuronal soma collects inputs from a multitude of synapses subject to individual (and rather independent) structural and functional plasticity processes. Synaptic plasticity is recognized to underlie learning and memory and, according to the synaptic plasticity and memory hypothesis, it is necessary and sufficient for the storage of information required for the type of memory mediated by the brain area in which that plasticity is observed (Takeuchi et al., 2014).

The gap between the whole-neuron and the synaptic scales opens the question about the existence of *synaptic* engrams, prompting the implementation of appropriate methods for the visualization, analysis and manipulation of individual synapses undergoing learning-related plasticity. Such methods are indeed starting to appear (Hayashi-Takagi et al., 2015; Gobbo et al., 2017), and are the subject of the Review by Gobbo and Cattaneo.

The possibility to affect an acquired behavior by specifically acting on the synapses potentiated during the learning phase of a task (Hayashi-Takagi et al., 2015) demonstrates that analyzing engrams at the whole-cell scale could be missing the finer details of the picture. The Original Research Article by Perez-Alvarez et al. presents experiments on organotypic brain slices using a custom-made microscope and a tubulin-bound version of the CaMPARI2 Ca^2+^ sensor, TubuTag, showing the existence of a dendritic shaft Ca^2+^ gradient in response to focal stimulation. Even though TubuTag cannot visualize individual dendritic spines, the results suggest a possible mechanism for the generation of clusters of potentiated dendritic spines, which have been also observed, for instance, in fixed mouse hippocampi (Gobbo et al., 2017).

Of course, clustering of potentiated synapses is not a trivial byproduct of Ca^2+^ (or potentiation-related protein) spillover out of the stimulated site, but represents an efficient way to implement the associativity and cooperativity rules of synaptic plasticity, as well as to integrate multiple synaptic inputs. The Review by Kastellakis and Poirazi describes the different faces of synapse clustering, how it can be achieved, and its relevance for the pathophysiology of brain circuits, with a careful analysis of its cellular-molecular basis, along with computational modeling studies aimed at extracting the rules for and the advantages of grouping potentiated synapses together.

Variations and rearrangements in the protein content of synapses provide the molecular basis for synaptic potentiation; at glutamatergic synapses, which mediate most of the brain's excitatory neurotransmission, the addition of new neurotransmitter receptor units to obtain an increase of synaptic currents (Diering and Huganir, 2018) is an evident example of molecular plasticity. However, there is much more to synaptic potentiation. By concentrating on PSD-95, researchers showed a remarkable diversity in its interactome, with large supramolecular complexes comprising cytoskeletal elements, adaptors, kinases and phosphatases (Fernandez et al., 2009). On the other hand, a full understanding of the relationship between protein composition and synapse functional state(s) is complicated by the heterogeneity of the synaptic repertoire of the brain, which is not taken into account by the vast majority of approaches for studying synapse composition (Grant and Fransen, 2020). A step toward this direction is the combination of regulatory sequences from the mRNA of the immediate early-gene *Arc* (as used by Hayashi-Takagi et al., 2015) with an oligopeptide enhancing synaptic localization to achieve *in vivo*, activity-dependent local translation at potentiated dendritic spines of genetically encoded reporters or actuators, such as channelrhodopsins or proteomic probes (Gobbo et al., 2017; Mainardi et al., 2019).

These state-dependent proteomic studies will ultimately provide the molecular fingerprint associated to information storage at potentiated synapses. The Hypothesis article by Goult speculates that a molecular code for memory storage exists, and describes a provocative possibility of a binary code centered around scaffolding proteins. Abundant experimental effort will be required to prove whether this speculative code actually exists.

An additional aspect in the elucidation of the neural basis for engram formation is the contribution of inhibitory (inter)neurons to memory-related synaptic circuits and cellular ensembles. Interestingly, inhibitory neurons express a repertoire of immediate-early genes closely matching excitatory neurons, including *cFos*. Moreover, different classes of inhibitory neurons appear to have specific roles in different phases of memory. Finally, GABAergic circuits are thought to participate in keeping memory traces latent. These important issues are discussed in the Review by Giorgi and Marinelli.

We are confident that the different contributions of this Research Topic provide an overview and a primer on the most relevant issues and unaddressed questions in memory engram research. We also believe that experimental efforts should concentrate on (i) defining the molecular identity of synapses in a state-specific manner (i.e., basal activity, short- and long-term potentiation); (ii) mapping the distribution of synapses potentiated during the different phases of a learning and memory recall task to provide reliable datasets for the elaboration of computational models; (iii) addressing the relationship between cellular memory engrams and *bona fide* synaptic engrams. All these points are relevant to basic neurophysiology, but might also provide a strong knowledge basis for a substantial advancement toward the elaboration of therapeutic approaches to developmental and neurodegenerative synaptopathies.

## Author Contributions

AC and MM wrote, revised the manuscript, and approved it for publication.

## Funding

This work was supported by the Grant PRIN 2017 2017HPTFFC from the Italian Ministry of University and Research to AC.

## Conflict of Interest

The authors declare that the research was conducted in the absence of any commercial or financial relationships that could be construed as a potential conflict of interest.

## Publisher's Note

All claims expressed in this article are solely those of the authors and do not necessarily represent those of their affiliated organizations, or those of the publisher, the editors and the reviewers. Any product that may be evaluated in this article, or claim that may be made by its manufacturer, is not guaranteed or endorsed by the publisher.
